# Strain and atomic stacking of bismuth thin film in its quasi-van der Waals epitaxy on (111) Si substrate

**DOI:** 10.1038/s41598-023-46860-z

**Published:** 2023-11-13

**Authors:** Chia-Hsuan Wu, Chieh Chou, Hao-Hsiung Lin

**Affiliations:** 1 Graduate Institute of Electronics Engineering, National Taiwan University, Taipei, 10617 Taiwan, ROC; 2 Department of Electrical Engineering, National Taiwan University, Taipei, 10617 Taiwan, ROC

**Keywords:** Materials science, Nanoscience and technology

## Abstract

We report on the structural properties of Bi thin films grown on (111) Si substrates with a thickness of 22–30 BL. HRXRD and EBSD measurements show that these Bi films are mainly composed of twinning grains in the (0003) direction. The grain size can be as large as tens of microns. From a double-peak (01$\overline{1}$4) φ-scan, we found two pairs of twinning phases coexisting with a rotation angle of ~ 3.6°. We proposed a coincidence site lattice model based on preferential close-packed sites for Bi atoms on Si (111) surface to explain the coexistence of the rotation phases in the quasi-van der Waals epitaxy. From the measured lattice constants c and *a* of our samples, along with the data from the literature, we derived a c–*a* relation: (c–c_0_) = − 2.038(*a*–*a*_0_), where c_0_ and *a*_0_ are the values of bulk Bi. The normalized position of the second basis atom in the unit cell x, in these strained Bi films is found very close to that of bulk Bi, indicating that the strain does not disturb the Peierls distortion of the lattice. The fixed ratio of bilayer thickness to lattice constant c, reveals that the elastic properties of covalent-bonded bilayer dominate those of Bi crystal.

## Introduction

Bulk bismuth is a unique semi-metal with a small band overlap and very small effective mass in certain orientations in its conduction bands and valence bands^1,2^. The unique properties make bismuth a versatile material that can be manipulated in between semi-metal and semiconductor through quantum size effect. Recent studies on ultra-thin bismuth films also revealed peculiar surface bands resulting from strong spin–orbit interaction^3–7^. These properties have drawn attention to Bi thin film, which is promising for functional electronic and magnetic devices.

**Figure 1:**
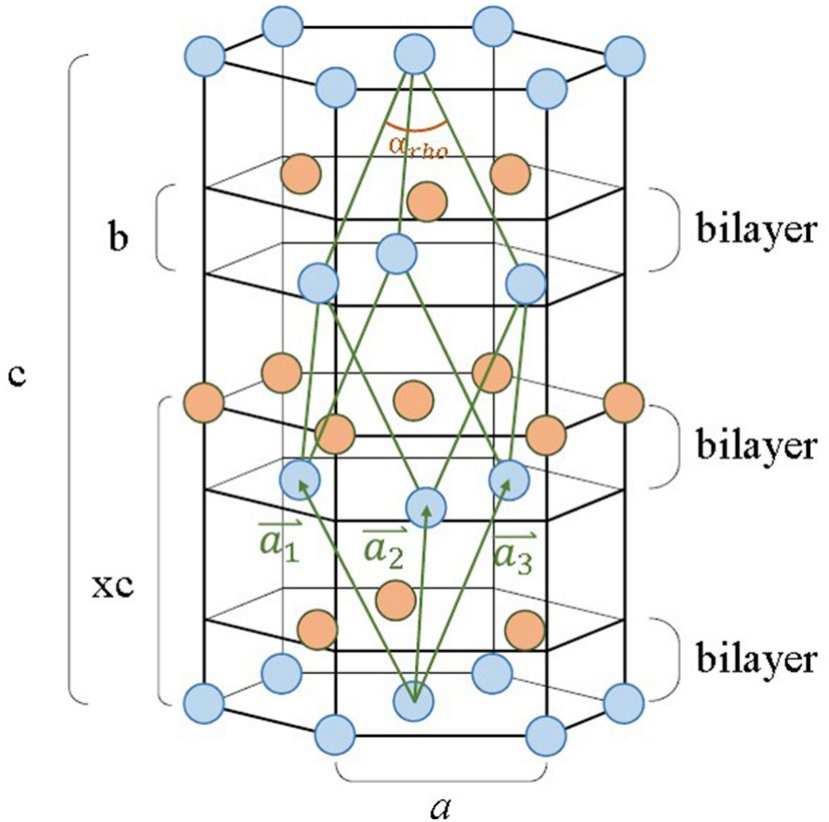
Schematic diagram of rhombohedral Bi lattice drawn in a hexagonal lattice. The two basis atoms are represented by light blue and light orange balls, respectively. Parameters b and x are the bilayer thickness and the normalized distance of the second basis, respectively.

The hexagonal lattice structure of bulk crystal Bi is shown in Fig. 1. The lattice shows three bilayers (BLs), stacking in ABC closed pack sequence along the trigonal direction (c-axis). In each bilayer, Bi atoms in the lower layer (in light blue) covalently bond three Bi atoms in the upper layer (in light orange), and vice versa. Bi atoms between the adjacent bilayers also have covalent charges and form a much weaker “semi-covalent” bonding^8^ or van der Waals bonding^9^. A green trigonal unit cell, containing two basis atoms, is also shown in the figure. The position of the second basis atom (in light orange) is normalized to the body diagonal of the unit cell c, and is denoted as x. The trigonal cell is distorted from a rock-salt cubic cell through a so-called Peierls-Jones mechanism^10–12^. The distortion shifts the normalized position x of the second basis atom from 0.5, the value for rock-salt lattice, destroying the cubic symmetry and dimerizing the atoms along the c-axis. Such a distortion introduces a small band gap to stabilize the system energy. However, the stable x position is liable to be perturbed by external influences^11^. The relief of Peierls distortion by highly injected electrons has been demonstrated^11,12^.

For the growth of Bi thin film on (111) Si substrates, Nagao et al*.* and Kammler et al*.* have demonstrated a method of preparing ultra-thin Bi films on a clean Si (111) 7 × 7 reconstruction surface at room temperature^8,13,14^. These previous works have utilized in-situ low-energy electron diffraction technique to observe a structure transition from textured pseudo-cubic Bi (110) grains into hexagonal Bi (111) grains when the coverage of Bi is about 5–7 monolayers. The Bi (111) grains have perfect azimuthal alignment with the Si (111) substrate with a relation of 6*a*_Bi_ = 7*a*_Si_. After the transition, the Bi (111) grains gradually coalesce and develop into a continuous layer.

In our previous work^15^, we found that the lattice constants of a ~ 80-nm-thick Bi film are all close to those determined from bulk Bi^16,17^, indicating that the growth is fully relaxed despite the large lattice mismatch between Bi and Si. In this work, we studied the properties of Bi thin film with thickness in the range of ~ 10 nm. Through high-resolution X-ray diffraction (HRXRD) measurements, the lattice parameters, c and *a*, were determined and the film was found strained. From the φ-scan of (01$\overline{1}$4) planes, we observed the coexistence of two phases with a small rotation angle. A coincidence site lattice model was proposed to explain the phenomena. The normalized position x of the second basis atom was also determined and was found unchanged.

## Results and discussions

**Figure 2:**
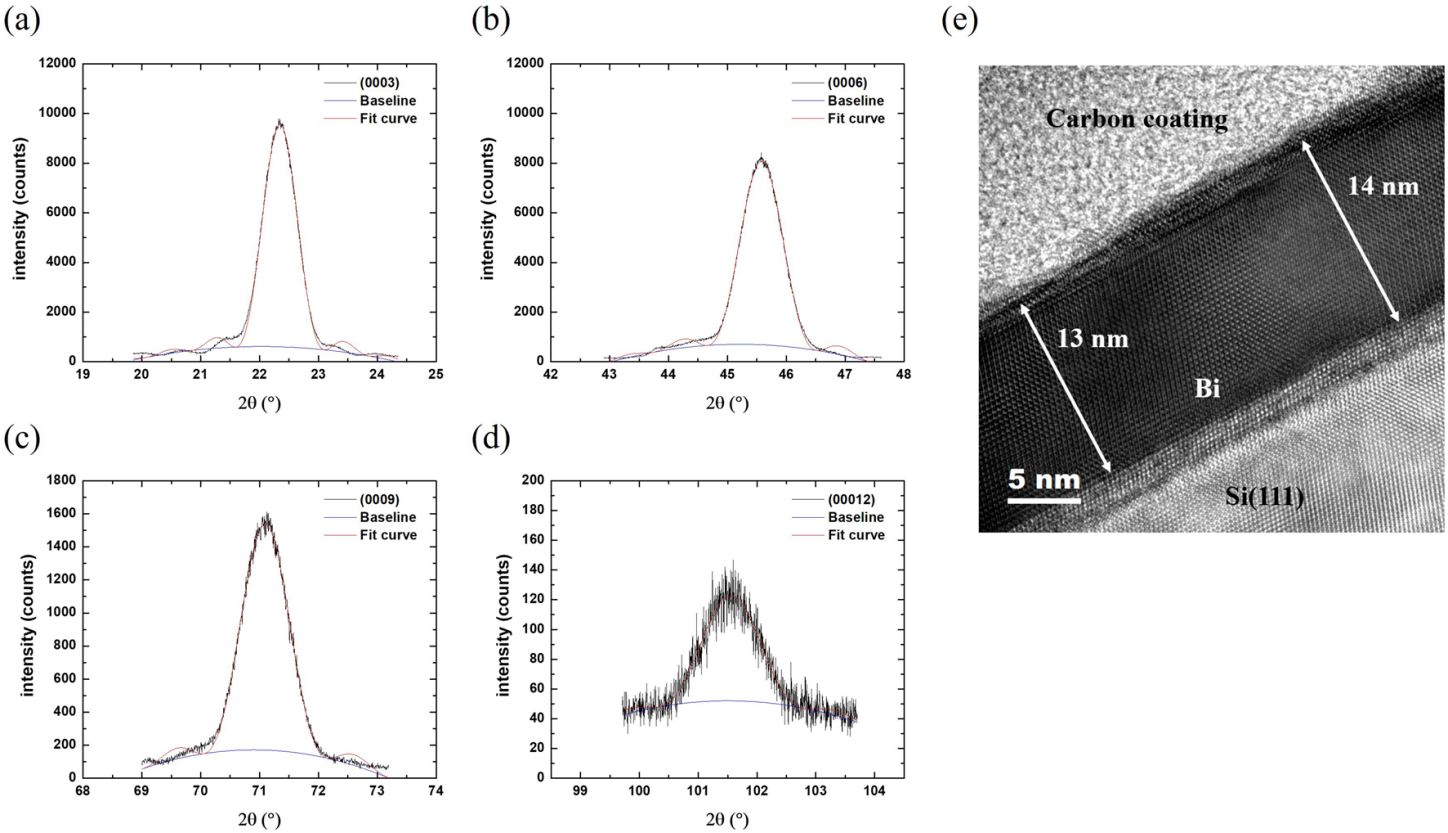
HRXRD Ω-2θ scans of (**a**) Bi (0003), (**b**) Bi (0006), (**c**) Bi (0009), (**d**) Bi (00,012) planes for sample S1. The red lines show the fitted results using Eq. (1). (**e**) HRTEM image of S1. The Bi film is comprised of 31–32 atomic layers, corresponding to a thickness of 12–13 nm.

Figure 2a–d show the Ω-2θ scans for (0003), (0006), (0009), and (00012) planes of sample S1, respectively. The linewidths of these peaks are broad, which is due to the thin thickness, i.e., Scherrer broadening^18^. We used an interference function for XRD to fit the line-shapes. The function^18^ is,

1 $$\begin{array}{*{20}c} {{\text{I}}_{{\text{p}}} \left( {\uptheta } \right) = {\text{I}}_{0} \frac{{\sin^{2} \left[ {{\pi M}\left( {\frac{{\sin {\uptheta }}}{{\sin {\uptheta }_{{\text{B}}} }}} \right)} \right]}}{{{\text{sin}}^{2} \left[ {{\uppi }\left( {\frac{{\sin {\uptheta }}}{{\sin {\uptheta }_{{\text{B}}} }}} \right)} \right]}}} \\ \end{array}$$

where θ is the angle between the incident X-ray and the plane, θ_B_ is the Bragg’s angle of the plane, M is layer number and I_0_ is a constant intensity. In addition to the line-shape function, the fitting also considered a parabolic background to reduce the effect of background noise, and the fitting results are summarized in Table 1. The average c is 11.932 Å, which is longer than the value reported for bulk Bi^16,17^, suggesting the existence of the vertical strain in the layer. The layer numbers, M’s, can be converted to the number of bi-layer (BL) to estimate the thickness. Figure 2e shows the cross-sectional TEM lattice image of the Bi thin film. The thickness obtained by TEM is 31–32 atomic layers, which is close to the 30 BL obtained by the interference function fitting for (0003) reflection. The SAED patterns of this sample (not shown) are similar to those of 80-nm-thick Bi film reported previously^15^. Detailed analysis about the epitaxial relationship between Bi and Si substrate will be provided in the subsequent HR-XRD φ scan experiments.

**Table 1:** Results of lattice parameters of S1, which are fitted by interference function.

Sample S1	2θ (°)	d (Å)	c (Å)	M (layer)	Integrated intensity
(0003)	22.342	3.977	11.931	30	8.95 × 10^3^
(0006)	45.581	1.989	11.934	52	7.38 × 10^3^
(0009)	71.095	1.325	11.927	78	1.36 × 10^3^
(00012)	101.554	0.995	11.935	113	7.05 × 10^1^

**Figure 3:**
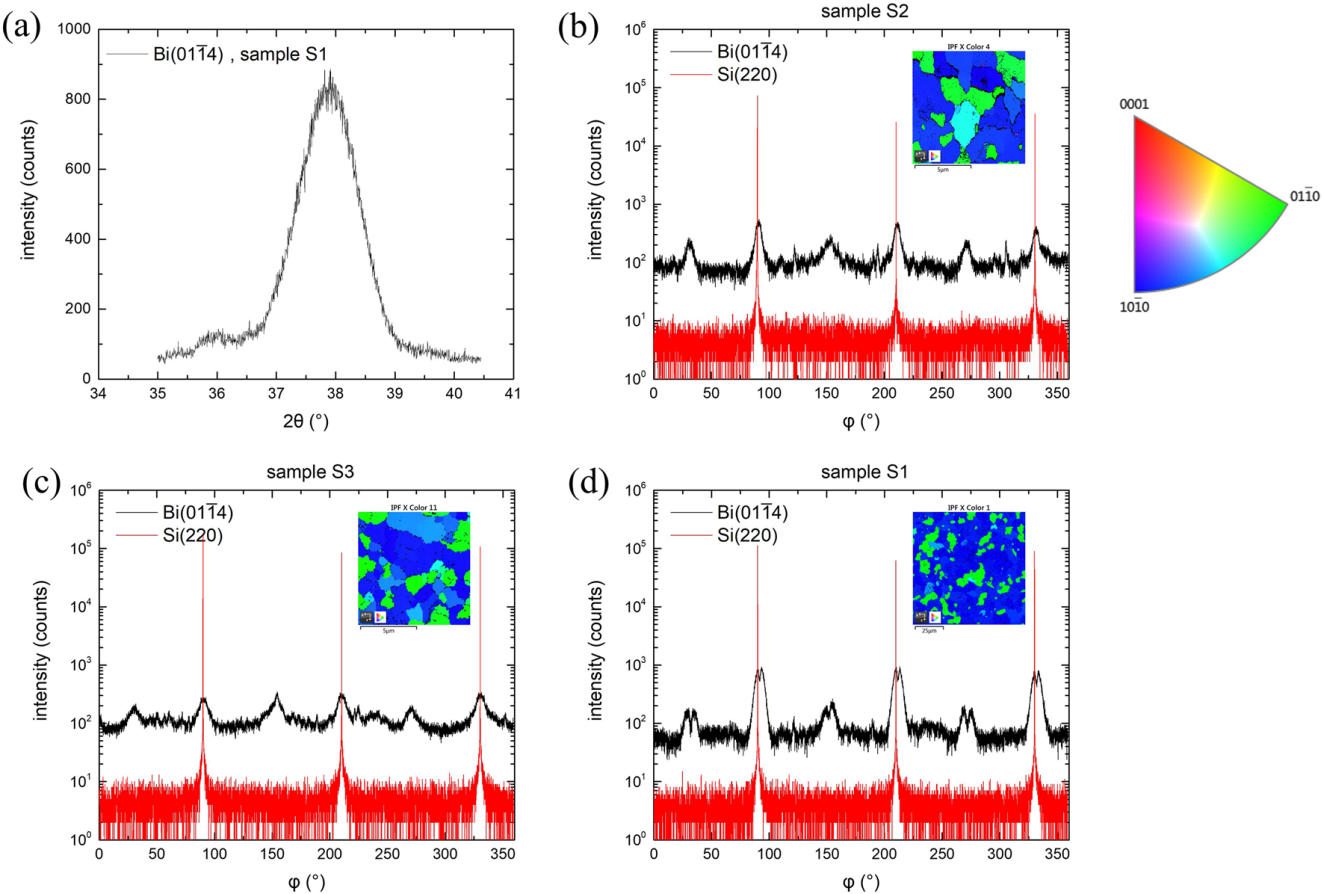
(**a**) A typical HRXRD Ω-2θ scan of tilted Bi (104) plane for sample S1. (**b**–**d**) HRXRD φ scans of Bi (014) and Si (220) planes for samples S2, S3 and S1 respectively. The EBSD inverse pole figures (IPF X) are shown as the inset in their panels. The crystal orientation is indicated by the colored sector on the right of (**b**).

To study the in-plane structures, we performed XRD measurements on tilted plane (01$\overline{1}$4). A typical Ω-2θ scan for the tilted plane (01$\overline{1}$4) is shown in Fig. 3a, and the (01$\overline{1}$4) φ-scans of three Bi samples are shown in Fig. 3b–d. For comparison, the (220) φ-scan of their Si substrates is also shown in the figures. Detailed procedures for XRD measurements on tilted planes have been reported previously^15^. Lattice constant *a* can be resolved from the following equation,

2 $$\begin{array}{*{20}c} {\begin{array}{*{20}c} {\frac{1}{{{\text{d}}_{{\left( {{\text{hklm}}} \right)}}^{2} }} = \frac{4}{3}\frac{{{\text{h}}^{2} + {\text{hk}} + {\text{k}}^{2} }}{{a^{2} }} + \frac{{{\text{m}}^{2} }}{{{\text{c}}^{2} }}} \\ \end{array} } \\ \end{array}$$

where d_(hklm)_ is the thickness of the plane (h k l m), and h = 0, k = 1, and m = 4 for plane (01 $\overline{1}$ 4). We proceeded series of (01$\overline{1}$4) measurements, the results of *a* are listed in Table 2. The average *a* of Bi films are all smaller than the reported values for bulk Bi^16,17^.

**Table 2:** Lattice parameter c and *a*.

	c(Å)	*a*(Å)	Thickness	Grain size	Method
Barrett^17^	11.862 ± 0.001	4.546 ± 0.0002	Ingot	Single crystal	XRD
Nagao^8^	4.0 ± 0.2^a^	4.480 ± 0.01	> 6 ML	–	STM, SPA-LEED
Shirasawa^19^	11.96 ± 0.04	4.50 ± 0.02	6 nm	–	Surface XRD
Hirahara^7^	1.64 ± 0.04^b^ 2.42 ± 0.04^c^	4.39 ± 0.05	6 BL	–	SPA-LEED
Chou^15^	11.870 ± 0.003	4.545 ± 0.01	80 nm	~ 2 μm	XRD (D8)
This work S1	11.932 ± 0.0015	4.519 ± 0.01	12 nm	12.5 μm	XRD (D8)
This work S2	11.906 ± 0.022	4.530 ± 0.01	10 nm	~ 2 μm	XRD (D8)
This work S3	11.944 ± 0.005	4.508 ± 0.02	9 nm	~ 1 μm	XRD (NSRRC)
This work S4	11.953 ± 0.005	4.505 ± 0.02	11 nm	~ 2 μm	XRD (NSRRC)

^a^Monolayer thickness, equal to c/3, which is obtained from Fig. 2k of Ref^8^.

^b^Intrabilayer thickness, equal to b.

^c^Interbilayer thickness, equal to (c/3)−b.

Figure 3b shows the (01$\overline{1}$4) φ-scan of sample S2. As shown in the figure, there are three stronger peaks interleaved by three weaker peaks. Note that the (01$\overline{1}$4) plane is with threefold symmetry. The stronger 3 (01$\overline{1}$4) peaks belong to a twinning phase resembling the stacking sequence of the Si substrate, and the φ angles are aligned to those of Si (220) with an angle difference of ~ 1.4° only. The weaker 3 peaks belong to the other twinning phase. The lower intensity suggests that it is less preferential than the former one, which is supported by the EBSD IPF X image shown as the inset of the figure. In Fig. 3c, the φ-scan of sample S3 is shown, the six (01$\overline{1}$4) peaks are in the same intensity level, indicating that the two twining phases are with equal preference. However, three of the peaks well match the (220) peaks of the Si substrate, with an angle difference of less than 0.1°. The average grain sizes of sample S2 and S3, estimated from their EBSD IPF X images are 1 ~ 2 μm. The (014) φ-scan of sample S1, whose average grain size is in tens microns, is shown in Fig. 3d. We can see that all the six (01$\overline{1}$4) signals split into two peaks, suggesting that in addition to the two twining phases, there appears a pair of misaligned twinning phases in this sample. The Si (220) peaks well align the left split peaks of the primary twining peaks, and the right split peaks are ~ 3.6° apart from the Si (220) peaks. For the other twinning phase, the angle difference between the split peaks is ~ 5°.

**Figure 4:**
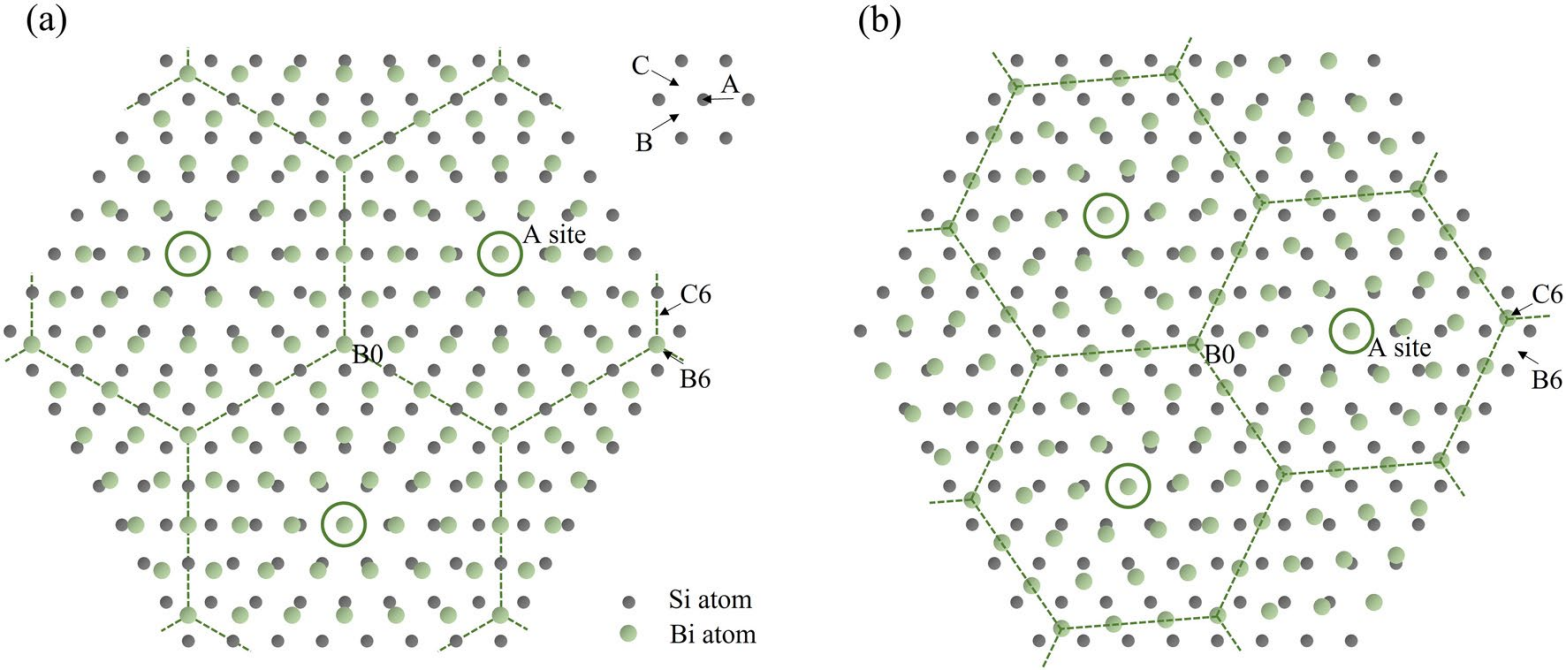
(**a**) Sketch of Bi atomic arrangement that satisfies 6*a*_Bi_ = 7*a*_Si_. The Bi atoms form a $2\sqrt 3 \times 2\sqrt 3$ (*a*_Bi_) supercell indicated by a dashed hexagon. (**b**) Sketch of the misaligned atomic arrangement. The Bi atoms form a $3 \times 3$ (*a*_Bi_) supercell indicated by a dashed hexagon.

To explain the coexistence of the two Bi grains, we consider the atomic stacking at the Bi/Si interface. Figure 4a shows an atomic arrangement based on the 6 Bi to 7 Si registry reported previously^13,14^. In this model, we assume that 6*a*_Bi_ = 7*a*_Si_ and the preferential sites for Bi atoms are the A, B, and C sites of the close-packed hexagonal lattice of the Si (111) surface layer. A site is the position right on top of Si surface atoms, and B and C sites are the other two possible sites for close-packed hexagonal layer. Notice that Bi atom has two different bonds, i.e., covalent and semi-covalent bonds, with different bond lengths in Bi crystal. This behavior could allow Bi atoms to stand directly on top of Si atom (A site) or to occupy the other two close-packed sites (B site and C site). As shown in the figure, the Bi atoms sitting on A, B, or C sites form a $2\sqrt 3 \times 2\sqrt 3$ (*a*_Bi_) supercell, indicated by a dashed hexagon. The hexagon has its six corner atoms alternately fall in the B-site and the C-site of the Si lattice, and the central atom is at the A-site. Note that the Bi atoms on A-site form a 6 × 6 (*a*_Bi_) coincidence site lattice with the bottom Si 7 × 7 (*a*_Si_) lattice. Figure 4b shows the misaligned atomic arrangement. In this model, the Bi lattice is slightly rotated to shift the sixth Bi atom from the origin preferential site B6 of the well registry model (Fig. 4a) to the nearest preferential site C6. The lattice constant *a*_Bi_ is slightly extended to 6*a*_Bi_ = 7.024*a*_Si_, and the rotation angle is ~ 4.7°. As shown in the figure, the Bi atoms sitting on A, B, or C sites form a 3 × 3 (*a*_Bi_) supercell, indicated by a dashed hexagon. Like the case in Fig. 4a, the hexagon has its six corner atoms alternately fall in the B-site and the C-site of the Si lattice, and the central atom is at the A-site. The Bi atoms on A-site form a 3 $\sqrt 3 \times 3\sqrt 3$ (*a*_Bi_) coincidence site lattice at the Bi/Si interface, and the bottom Si coincidence site lattice is $\sqrt {37} \times \sqrt {37}$ (*a*_Si_). Yaginuma et al*.* suggested that the formation of 6 × 6 (*a*_Bi_) and Si 7 × 7 (*a*_Si_) coincidence site lattice is the key step for the following highly crystallized Bi lattice^13^. They used DFT theory to calculate the *a*_Bi_ of freestanding Bi slab with a thickness ranging from 1 to 8 hexagonal bi-layer. When the thickness reaches 3 bilayers, where the hexagonal nuclei stabilize on the Si substrate, the calculated *a*_Bi_ = (7/6) *a*_Si_, in good agreement with the value obtained from SPA-LEED measurement. This lattice-match information is then conveyed to the growth film through the hexagonal nuclei. We believe that in addition to the 6 × 6 (*a*_Bi_) coincidence site lattice shown in Fig. 4a, the 3 $\sqrt 3 \times 3\sqrt 3$ (*a*_Bi_) coincidence site lattice, shown in Fig. 4b plays the same role. In fact, Kammler et al*.* have mentioned the observation of a second preferred Bi grain with a 4.7° rotation to the direction of the main Bi grain when the Bi coverage is 7 ML^14^. In this early stage, their reported rotation angle is almost matched to the value predicted by the models we proposed.

The thickness of our samples is within 25–30 BL. These films have undergone grain coalescence into a continuous layer, and may have begun to relax. In coalesce process and the following relaxation process, shear stress resulting from the formation of grain boundaries or relaxation could further rotate the in-plane orientation. As a result, the angles we observed are slightly different from the predicted values. We have tried other misalign arrangements by shifting the sixth Bi atoms from the B6 site to other adjacent B-sites or C-sites, and found that the lattice constants *a*_Bi_ of these cases deviate from that of the well-registered case at least 4.9%, and thus unlikely to be the cases for Bi lattices.

**Figure 5:**
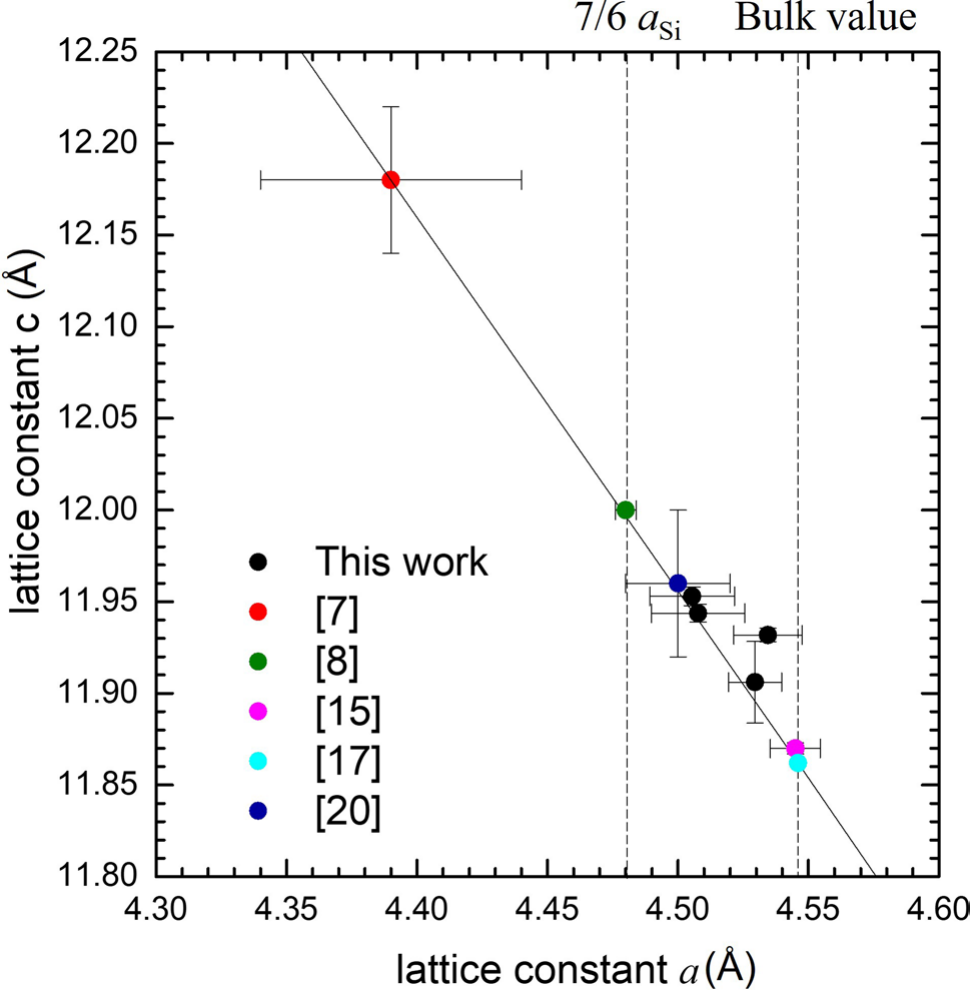
Summary of Bi lattice constant *a* and c for sample S1–S4, along with the data reported in the literature^7,8,15,17,19^. The solid line satisfies the c-*a* relation (c/c_0_−1) = − 0.781(*a*/*a*_0_−1).

Figure 5 shows the relationship between lattice constants c and *a* for our four samples along with several data reported in the literature^7,8,15,17,19^. Except for the 6-BL-thick Bi film grown on Bi_2_Te_3_ substrate^7^, all the Bi thin films grown on Si substrate are within the left and right vertical dotted lines, which represent *a*_Bi_ = 7*a*_Si_/6 = 4.480 Å and *a*_Bi_ = 4.546 Å, respectively. The former is the value reaching the coincidence lattice matched to Si substrate in the nuclei stabilizing stage^13^, which can be regarded as the starting point of the growth. While the latter is the bulk value reported by Barrett^16,17^, which was obtained from zone-refined single crystal ingot, free from strain. Therefore, it can be regarded as the end of the growth. As can be seen in the figure, there is an empirical solid line, and almost all the points are close to or on this line. On the assumption of biaxial strain, the line can be represented by the following equation:

$$\frac{{\varepsilon_{zz} }}{{\varepsilon_{xx} }} = \frac{{\left( {c/c_{0} } \right) - 1}}{{\left( {a/a_{0} } \right) - 1}} = - \frac{{2{\text{C}}_{13} }}{{{\text{C}}_{33} }}$$

where ε_zz_ is strain along trigonal axis, ε_xx_ is in-plane strain, c_0_ and *a*_0_ are the fully relaxed or freestanding lattice constants, and C_ij_’s are six Voigt stiffness constants^20^. In here, c_0_ and *a*_0_, are selected to be the values of Barrett, and the strain ratio − 2C_13_/C_33_ derived from the solid line is − 0.781, which is deviated from the values − 1.286, calculated from Eckstein’s stiffness constants^20^, and − 0.960, calculated from Bridgman’s elastic constants^21^. Both Eckstein’s constants and Bridgman’s constants were obtained from bulk Bi crystal bars.

According to Yaginuma et al*.* DFT calculation, the *a*_Bi_ of freestanding Bi slab increases with the thickness and gradually approaches to bulk Bi’s value^13^. Their calculation was from 1 to 8 BL, and the corresponding “strain” with respect to bulk *a*_Bi_ was from − 5.3 to − 0.8%. In here, strain needs a clear definition. The strain with respect to bulk’s *a*_Bi_ is called “apparent strain”. And the strain resulting from stress is called “effective strain”, and is defined as the strain with respect to the thickness-dependent freestanding *a*_Bi_. Different freestanding lattice constants may result in different c-*a* relations. Since the solid line in Fig. 5 contains Bi films with different thicknesses ranging from 6 to 200 BL, we assume that all the freestanding points stand on the same line. For the Bi film grown on Si substrate, in the early nuclei stage, the freestanding *a*_Bi_ of the nuclei makes the coincidence site lattice match to Si lattice. However, as the film thickness increases, the increasing freestanding *a*_Bi_ builds up effective strain and stress. Consequently, the strain relaxation moves the lattice constants of the film toward the freestanding *a*_Bi_ along the solid line. Although our results follow the solid line, their positions are not in order of thickness, which could arise from the complex granular structures and the internal atomic deviation, especially of the atoms not at the preferential sites in the lattice. In ref.^8^, Nagao et al*.* calculated the cohesive energy of hexagonal bilayer. From their result, when the thickness is larger than 8 BL, the cohesive energy per atom has been below 25 meV. For the growth close to room temperature, it is easy for Bi atoms to deviate from the lattice positions. The deviation may induce lattice distortion, which not only causes extra effective strain, but also enlarge the uncertainty of measured *a*_Bi_.

In the Bi unit cell shown in Fig. 1. The diagonal along the c-axis contains three BLs. In each BL, the atoms in the top and bottom layer belong to different basis atoms, and the bilayer thickness, b = c (x−1/3). The normalized basis position x affects the structure factor of the lattice and thus the integrated XRD intensity. By comparing the integrated XRD intensities of different (0 0 0 m) planes, one can find x. However, the integrated intensity is also a function of Bragg’s angle, θ, and Debye–Waller factor, e^−2M^. In here, we consider only the terms relevant to the normalized position, x, Bragg’s angle, θ, Debye–Waller factor, e^−2M^, and the Miller indices of the (0 0 0 m) plane, the integrated XRD intensity, I, can be expressed as follows,

$${\text{I}} = {\text{Ae}}^{{ - 2{\text{M}}}} {\text{Lf}}_{{{\text{Bi}}}}^{2} \cos^{2} \left( {\pi {\text{xm}}} \right)\left( {1 - {\text{e}}^{{ - \frac{2\mu t}{{\sin \theta }}}} } \right)$$

where A is a constant independent of Bragg’s angle and orientation, L is the Lorentz factor, f_Bi_ is the atomic scattering factor, μ is the absorption coefficient, and t is the layer thickness. The factor M of Debye–Waller factor can be expressed as,

$${\text{M}} = 8\pi^{2} \overline{{{\text{r}}_{{\text{z}}}^{2} }} \left( {\frac{\sin \theta }{\lambda }} \right)^{2} = {\text{B}}_{{\text{z}}} \left( {\frac{\sin \theta }{\lambda }} \right)^{2}$$

where λ is the wavelength of the X-ray, $\overline{{r_{z}^{2} }}$ is the average of the mean square atomic displacement along z direction and ${\text{Bz}} = 8\pi^{2} \overline{{{\text{r}}_{{\text{z}}}^{2} }}$. For the new D8 HRXRD system, the X-ray source is Cu-Kα and has a Ge (220) first crystal, the Lorentz factor, L, includes the polarization factor and the angular velocity factor and is given by

$${\text{L}} = \frac{{1 + \cos^{2} 2\theta_{{\text{M}}} \cos^{2} 2\theta }}{\sin 2\theta }$$

where θ_M_ is the Bragg’s angle for Ge (220) and cos 2θ_M_ = 0.7033. The absorption coefficient of Bi, μ, is 2391/cm. For the measurement at the TPS09A line, the X-ray source is horizontally polarized and with a photon energy of 13.3 keV. The Lorentz factor contains only the angular velocity factor, and L = 1/sin2θ. The absorption coefficient, μ, is 660.3/cm.

Finally, we divide Eq. (1) by the measured X-ray intensity, I_exp_, and define it as F(x),

$${\text{F}}\left( {\text{x}} \right) = \frac{I}{{I_{\exp } }} = \frac{{{\text{Ae}}^{{ - 2{\text{M}}}} {\text{Lf}}_{{{\text{Bi}}}}^{2} \cos^{2} \left( {\pi {\text{xm}}} \right)\left( {1 - e^{{ - \frac{2\mu t}{{\sin \theta }}}} } \right)}}{{I_{\exp } }}$$

**Figure 6:**
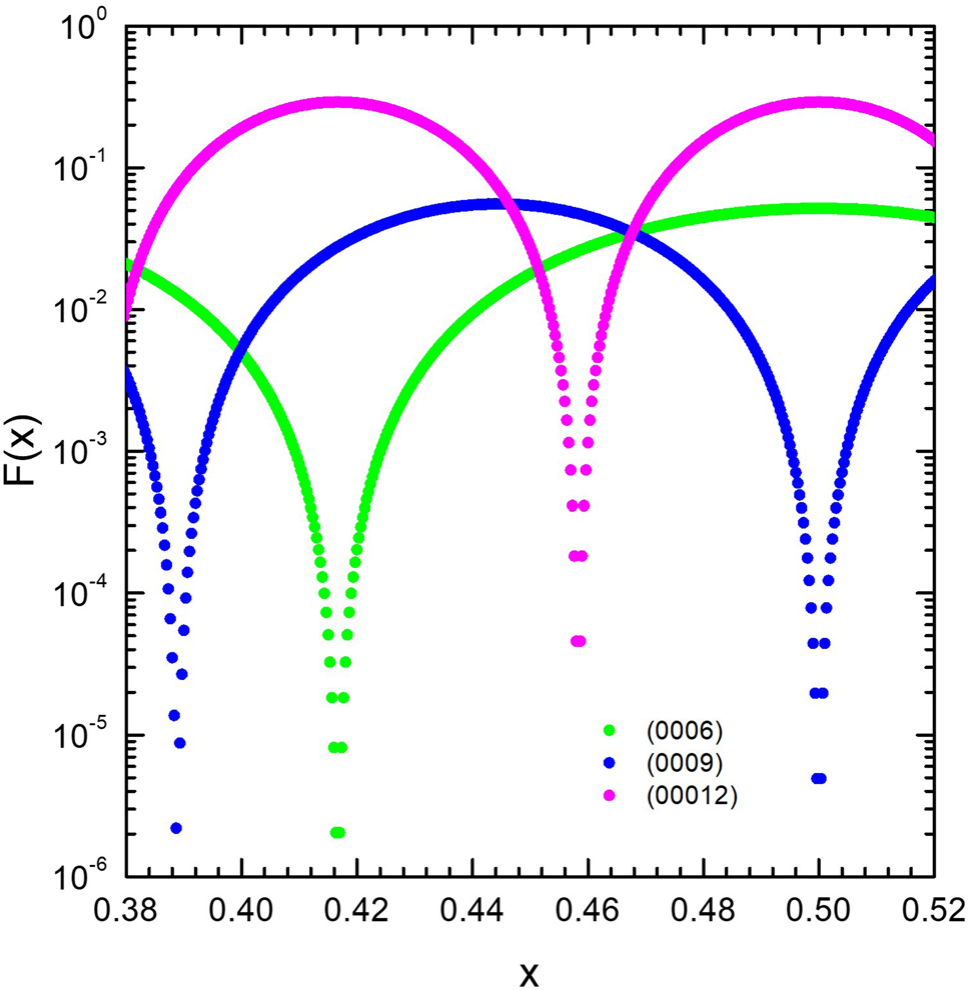
Logarithmic plot of F(x) versus x of the (000 m) planes for sample S1.

The F value should equal to A for all planes if we select the correct values for x and M. Since A, x and M are three unknown variables, at least three planes are needed to solve them. In here, (0006), (0009) and (00012) planes to resolve the problem. (0003) plane was not chosen because of the extinction effect resulting from its small Bragg’s angle^16^. For sample S1, a plot of F(b/d)/A as a function of x is shown in Fig. 6. The three curves intersect at a point at x = 0.4677 and the solved Bz = 3.012 Å^2^. The method was applied to other samples, and the results are summarized in Table 3. Compared with previous thicker films of about 80 nm in the laboratory^15^ and bulk materials reported in^16^, the difference in x is within 0.2%, showing that the strain does not affect the x value. In Fig. 4, the Bi film on Bi_2_Te_3_^7^ has the largest strain. The apparent strain is as large as − 3.3%. In the reference, Hirahara et al*.* also gave both intra-bilayer thickness, b, and inter-bilayer thickness, c/3−b, using LEED technique^7^. The x value calculated from the two thicknesses is 0.4680, in very good agreement with the bulk value as well as our values, listed in Table 1. value. Note that x value indicates a stabilization of the Peierls distortion applied to a rock-salt lattice (x = 0.5), which introduces a small band gap over the extended region of the Brillouin zone. Disturbing the stabilization by high electronic excitation has been reported. Our findings suggest that the strain does not affect the x, implying that the change of band gap structure by thickness reduction does not affect the stabilization, even to a thickness of 6 BL. A fixed value of x also implies a fixed relationship between b and c. It also means that the elastic properties of Bi crystal are dominated by the elastic properties of the covalently bonded bilayer network. The inter-bilayer semi-covalent bonds play a minor role. Its long-range effect causes a change in the freestanding lattice constant when the bilayer number is small.

**Table 3:** Lattice parameter x and B_z_.

	x	B_Z_(Å^2^)	Thickness
Barret^17^	0.4680	1.144	Ingot
Hirahara^7^	0.468^a^		
Chou^15^	0.4680	1.868	80 nm
This work S1	0.4677	3.013	12 nm
This work S3	0.4690	1.802	9 nm
This work S4	0.4680	2.249	11 nm

^a^Calculated from the intralayer and interbilayer thickness, also listed in Table 2.

## Conclusion

In conclusion, we have studied the structural properties of Bi thin films grown on (111) Si substrates with a thickness of 22–30 BL. The HRXRD and EBSD measurements showed that the epilayers are mainly composed of twinning grains in the (0003) direction and the grain size can be as large as tens of microns. In the HRXRD φ-scan of S1, we observed double-peaks with an angle difference of ~ 3.6°, suggesting the coexisting of two twinning phases. We proposed a coincidence site lattice model based on preferential close-packed sites for Bi atoms on Si (111) surface to explain the two phases in the quasi-van der Waals epitaxy. From the measured lattice constants c and *a* of our samples, along with the data from the literature, we derived a c–*a* relation: (c/c_0_−1) = − 0.781(*a*/*a*_0_−1), where c_0_ and *a*_0_ are the values of bulk Bi. The normalized position of the second basis atom in the unit cell, x, was also determined in these strained Bi films. We found that all the x values are very close to that of bulk Bi, indicating that neither the strain nor the geometric thin structure disturbs the Peierls distortion of the lattice. The fixed ratio of bilayer thickness to lattice constant c, reveals that the elastic properties of covalent-bonded bilayer dominate those of Bi crystal.

## Methods

### MBE of nanoscale Bi

An SVTA solid source molecular beam epitaxial system was used to grow the Bi thin films on n-type Si (111) substrates. The Si substrates were immersed in acetone, methanol, and isopropanol each for 2 min and then soaked in 2% HF solution for 1 min to remove native oxide. Afterward, the substrates were loaded into the MBE system and degassed at 300 °C for 1 h in the buffer chamber. For the growth, the substrates were transferred to the growth chamber and heated to 900 °C for 5 min to obtain 7 × 7 surface reconstruction^13,14,22^. The substrate temperature was then cooled down to 5 °C. The shutter of the Bi K-cell was opened for a growth of 5 min.

### HRXRD spectra

HRXRD spectra of Bi film were carried out on a Bruker New D8 Discover system. The X-ray wavelength is 1.5406 Å (Cu K-α). Two samples were measured at beamline TPS09A, National Synchrotron Radiation Research Center (NSRRC), in Hsinchu, Taiwan. The energy of the beam was 13.3 keV, and the spot size was 0.5 × 0.8 mm.

### HRTEM image

HRTEM measurements were carried out on JEOL JEM-2010F TEM with an accelerating voltage of 200 kV.

### EBSD maps

EBSD maps of Bi film were performed on JEOL JSM-7800F PRIME with EBSD NordlysMax3 detector. The accelerating voltage is 20 kV.

## Data Availability

The data sets used and analyzed in the current study are available upon reasonable request from the corresponding author.
